# Acceptance and Commitment Therapy to support medication decision-making and quality of life in women with breast cancer: protocol for a pilot randomised controlled trial

**DOI:** 10.1186/s40814-022-00985-6

**Published:** 2022-02-08

**Authors:** Samuel G. Smith, Rachel Ellison, Louise Hall, Jane Clark, Suzanne Hartley, Ellen Mason, Jamie Metherell, Catherine Olivier, Vicky Napp, Jay Naik, Sarah Buckley, Charlotte Hirst, Sue Hartup, Richard D. Neal, Galina Velikova, Amanda Farrin, Michelle Collinson, Christopher D. Graham

**Affiliations:** 1 Leeds Institute of Health Science, University of Leeds, Leeds, LS2 9JT UK; 2 Clinical Trials Research Unit, Leeds Institute of Clinical Trials Research, University of Leeds, Leeds, LS2 9JT UK; 3 Department of Clinical and Health Psychology, St James’s University Hospital, Beckett Street, Leeds, LS9 7TF UK; 4 Department of Oncology, Harrogate & District Foundation Trust, Park Road, Lancaster, HG2 7SX UK; 5 Department of Clinical Research, Mid Yorkshire Hospitals NHS Trust, Aberford Road, Wakefield, WF1 4AL UK; 6 St James’s University Hospital, Beckett Street, Leeds, LS9 7TF UK; 7 Department of Psychology, Queen’s University Belfast, Belfast, BT7 1NN UK

**Keywords:** Medication adherence, Breast cancer, Acceptance and Commitment Therapy, Pilot, Quality of life, Remote delivery, Group therapy, Video conferencing

## Abstract

**Background:**

Adherence to adjuvant endocrine therapy is affected by medication side-effects and associated distress. Previous interventions focused on educating women to enhance adherence have proved minimally effective. We co-designed an Acceptance and Commitment Therapy (ACT) intervention to enhance medication decision-making and quality of life by targeting a broader range of factors, including side-effect management and psychological flexibility. This study aims to establish key trial parameters, assess the acceptability of the intervention and the extent to which it can be delivered with fidelity, and to demonstrate “proof of principle” regarding its efficacy on primary and process outcomes.

**Methods:**

The ACTION intervention includes an individual 1:1 ACT session followed by three group sessions involving 8–10 women and two practitioner psychologists. Participants are also provided with access to a website containing evidence-based methods for self-managing side-effects. The ACT sessions were adapted during the COVID-19 pandemic to be remotely delivered via video conferencing software. To evaluate the feasibility and acceptability of this intervention, a multi-site, exploratory, two-arm, individually randomised external pilot trial with a nested qualitative study will be undertaken. Eighty women with early stage breast cancer prescribed adjuvant endocrine therapy will be randomised (1:1) to receive treatment as usual or treatment as usual plus the ACTION intervention. The planned future primary outcome is medication adherence assessed by the ASK-12 measure. Progression to a phase III RCT will be based on criteria related to recruitment and follow-up rates, acceptability to patients, competency and fidelity of delivery, and proof of principle for change in medication adherence.

**Discussion:**

This external pilot trial will be used to ascertain the feasibility of undertaking a future phase III RCT to definitively evaluate an ACT-based intervention to support medication taking behaviour and quality of life in women with early stage breast cancer on adjuvant endocrine therapy.

**Trial registration:**

ISRCTN: 12027752. Registered 24 December 2020, 10.1186/ISRCTN12027752

## Background

Breast cancer is the most commonly diagnosed cancer in women worldwide [1]. In the UK, there are approximately 55,000 cases occurring per annum, with 11,000 cancer-related deaths [2]. Most breast cancers are oestrogen receptor positive (ER+) tumours. Treatment with adjuvant endocrine therapy (AET) such as Selective Oestrogen Receptor Modulators (SERMs, e.g. tamoxifen) and Aromatase Inhibitors (AIs e.g. letrozole, anastrozole, exemestane) for 5–10 years is standard care for women with ER+ tumours [3]. However, non-adherence is common with up to three-quarters of women prescribed adjuvant endocrine therapy (HT) taking < 80% of prescribed therapy and similar proportions discontinuing prematurely [4]. Low adherence and early cessation of AET are associated with an increased risk of breast cancer recurrence and all-cause mortality [5–12].

A broad range of behavioural and psychological variables such as mood, illness and treatment beliefs, and motivation are associated with adherence [13–15]. Medication side-effects are also frequently reported to affect adherence to HTs [4]. Common side-effects include hot flushes and night sweats, which affect up to 85% of women treated for breast cancer [16], and can compromise quality of life [17, 18]. Several other frequently reported medication side-effects may also affect adherence to HTs, including joint pain, vulvovaginal symptoms and sleep disturbance [19]. Most symptoms occur in the first year of using HT, suggesting this period could be the optimal opportunity for delivering supportive care for this patient group [20–22].

Adherence interventions evaluated so far have largely been ineffective [23–25]. A meta-analysis of 8 such interventions yielded a small to moderate heterogenous effect in favour of the interventions [24]. The interventions largely focussed on educating patients regarding AET and did not consistently address the range psychological factors that contribute to non-adherence. Broadening the focus to target emotions and self-management of side-effects could support adherence and quality of life in women who struggle with adhering to hormone therapy.

Acceptance and Commitment therapy (ACT) is a type of cognitive behaviour therapy that uses techniques such as mindfulness, goal setting, and perspective taking to engender psychological flexibility: “ … the capacity to persist or to change behaviour in a way that includes conscious and open contact with thoughts and feelings, appreciates what the situation affords, and serves one’s goals and values” [26]. ACT may be a suitable approach in this setting for two main reasons [13, 27–30]. First, psychological flexibility is a broad treatment target comprising a range of emotional and motivational factors that may contribute to non-adherence. Second, ACT is suitable to helping people with health conditions, largely because it does not focus on changing understandable emotional responses to illness (e.g. frustration, anxiety etc.). Instead, ACT encourages finding ways to live well alongside these difficult experiences to enable engagement in meaningful everyday activities [28, 31].

We propose that ACT could be used to support functioning and medication adherence with women who have experienced a life-threatening event such as breast cancer, and are being asked to return to everyday life while managing treatment-related challenges. There is a growing evidence base supporting ACT as a means to improve functioning, quality of life, and mood in chronic disease and pain [28, 32]. Although all have methodological limitations, several ACT trials with cancer populations have noted post-intervention improvements in outcomes, such as psychological distress and quality of life [28, 33–36]. There are no definitive trials of ACT for improving adherence to medication, with evidence limited to case series among people with diabetes and HIV [29, 37, 38].

We co-designed an intervention based on ACT principles through extensive engagement with patients and healthcare professionals [39]. The aim was to co-design an intervention that was acceptable to patients and implementable within the United Kingdom (UK) National Health Service (NHS). In patient focus groups we explored women’s experience of AET, perceptions regarding the acceptability of ACT, and preferences for intervention format. Healthcare professional interviews explored the acceptability and feasibility of an ACT intervention delivered within the NHS. In a co-design workshop, attended by patients and healthcare providers, we co-designed the configuration of the intervention. This process occurred prior to the coronavirus disease 19 (COVID-19) pandemic. We altered the co-designed intervention so that it could be delivered via video-conferencing software instead of in person. The aim of the adaption was to ensure the ACTION intervention could be delivered in the context of potential future changes in how clinical psychology services will provide psychological interventions, and to ensure it could be delivered while social distancing guidelines were in place for a clinically vulnerable population.

The aim of the ACTION trial is to test the feasibility of undertaking a definitive phase III parallel groups randomised controlled trial (RCT) of an ACT-based intervention for improving adherence to hormone therapy in women following curative treatment for breast cancer.

The objectives are to:

i) Establish eligibility, recruitment, retention and follow-up rates to inform the design of a phase III RCT;
ii) Assess the acceptability of the intervention and protocol to participants and NHS therapists;
iii) Assess the extent to which NHS therapists can remotely deliver an ACT intervention with fidelity, following remotely delivered training;
iv) Demonstrate “proof-of-principle”, via exploration of between-group change in outcomes (medication adherence, quality of life, mood) and process (psychological flexibility) variables.

## Methods

### Design

This is a multi-site, exploratory, two-arm, individually randomised pilot RCT with a nested qualitative study. Eighty women with early stage breast cancer will be randomised (1:1) to receive treatment as usual or treatment as usual plus the ACTION intervention. The study has been approved by the York and South Yorkshire Health Research Authority Research Ethics Committee (20/YH/0104), and is a registered clinical trial (ISRCTN12027752). The study is presented according to recommendations of the SPIRIT [40] and CONSORT extension for pilot and feasibility trials [41] (Appendix 1). The intervention being evaluated is described using the TIDieR checklist [42] (Appendix 1).

### Setting

We will identify and recruit participants from oncology services at NHS hospitals in Yorkshire, UK. Sites with existing or planned implementation of a psychological intervention designed to improve adherence to HTs will be excluded. A single site will be responsible for delivering the intervention (Leeds Teaching Hospitals NHS Trust). Eligibility criteria for the delivery site include access to a Health and Care Professional Council (HCPC) registered practitioner psychologist (Clinical, Health or Counselling Psychologist) to deliver the intervention alongside at least one additional supporting member of staff (e.g. psychologist in training, assistant psychologist, counsellor, nurse therapist, or other HCPC registered staff). The delivery site must also have access to video conferencing software with the ability to create virtual breakout rooms, which can accommodate groups of 8–10 individuals.

### Participants

Women aged over 18 and using tamoxifen, raloxifene, anastrozole, letrozole, or exemestane as part of their adjuvant endocrine therapy for early stage (1 to 3a) breast cancer are eligible. Full eligibility criteria are shown in Table 1.

**Table 1:** Eligibility criteria for participation in the ACTION trial

1. Written (signed and dated) informed consent 2. Capacity to provide informed consent 3. Women with early stage (1 to 3a) breast cancer according to the TNM/American Joint Committee on Cancer (AJCC) staging system 4. Aged ≥ 18 years at time of screening for ACTION 5. Have sufficient proficiency in English to contribute to the therapy sessions and data collection required 6. Treated with curative intent 7. Completed their hospital-based treatment (e.g. surgery, radiotherapy and/or chemotherapy). Women are still eligible for the study before completing Herceptin. 8. Currently prescribed oral adjuvant Hormone Therapy (tamoxifen, raloxifene, anastrozole, letrozole, exemestane) 9. The participant is willing to be audio recorded during the therapy sessions 10. The participant is willing to complete the study questionnaires 11. The participant is willing and able to attend all intervention sessions and/or complete therapy workbook 12. The participant is willing and able to access all sessions remotely via video call	1. Stopped taking adjuvant hormone therapy if it is clinically contraindicated according to clinical recommendation 2. Currently or recently (last 6 months) involved in another psychotherapy (e.g. using CBT/ACT, mindfulness) research study where medication adherence is a primary outcome 3. Those who are unable to access the sessions remotely via a video call 4. Currently attending, or on a waiting list for psychotherapy/psycho-oncology/psychology/counselling services, for any reason (related to medication or not) 5. Current diagnosis of an active major mental health disorder likely to interfere with participation (e.g. active psychosis, significant issues with addiction or self-harm) 6. Known element of risk (e.g. clinical team are aware that patient has made a recent attempt to end their life, or has recently disclosed plans to do so) as determined by three clinical screening questions below, (i) Recently (in the last month), have you had any thoughts about ending your life? * (ii) Have you thought about how you might go about it? (iii) Do you intend to carry out this plan? * a patient is only excluded if they answer ‘yes’ to question 6 (iii)

### Study processes

**Fig. 1:**
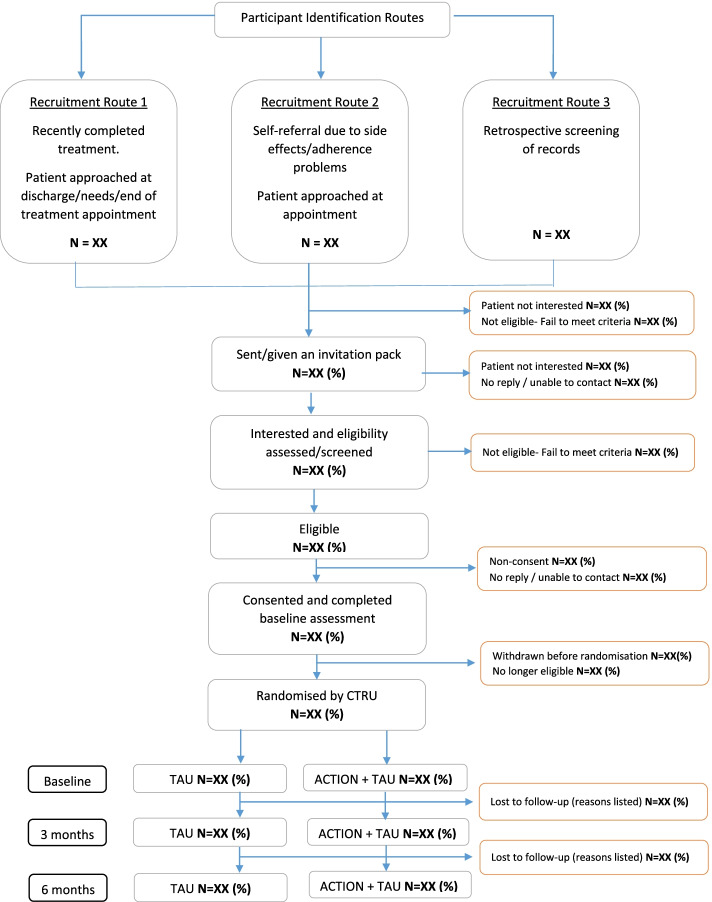
CONSORT diagram illustrating participant flow through the ACTION trial

Participant identification can be via three routes (Fig. 1):

1) A research nurse (or delegate) will screen patient records for eligible patients attending their final hospital appointment following completion of their hospital-based treatment;

2) An oncologist will screen patients discharged following treatment who self-refer to discuss problematic medication side effects and/or adherence problems;

3) A research nurse (or delegate) will retrospectively screen patient records for potentially eligible patients who have completed treatment within the last 6 months.

Sites will complete a screening form for all patients who have completed their hospital-based treatment for early stage breast cancer. The screening form will capture age, staging, ethnicity, and whether or not a patient is randomised. Screened patients who are not randomised will have the reason they are not eligible or declined participation recorded. Screening forms will not contain identifiable information.

For recruitment routes 1 and 2, a member of the patients care team will introduce a research nurse (or delegate) to the patient. For recruitment route 3, the research nurse or delegate will contact the patient by post. For all routes, a study invitation pack will be provided containing a participant information sheet, contact details, consent form, and a baseline questionnaire. Patients will have the opportunity to consent after considering the opportunity and asking questions, and may consent immediately once they are screened and confirmed as eligible. A research nurse will confirm eligibility among interested women, record consent, and ask the participant to complete the baseline questionnaire. Eligibility and consent discussions, and completion of baseline measures may take place in person at the hospital site or remotely. The site Principal Investigator (or delegate) is responsible for the informed consent of participants at their site.

Following informed consent, confirmation of eligibility and receipt of the baseline questionnaire, participants will be randomised by the research site using the University of Leeds Clinical Trials Research Unit (CTRU) randomisation system. Participants will be randomised on a 1:1 basis to receive either treatment as usual or treatment as usual plus the ACTION intervention and will be allocated a trial number. A computer-generated minimisation programme that incorporates a random element will be used to ensure treatment groups are well-balanced for the following stratification factors: recruiting site, recruitment route (recently completed treatment vs. experiencing medication problems vs. retrospective screening), participant age (≤ 50 years vs. > 50 years and < 70 years vs. ≥ 70 years).

Participants, therapists, research nurses/delegates at site, participant’s GP and staff at the Clinical Trials Research Unit (CTRU) will be not be blinded to the randomised allocation. Following randomisation the research nurse will notify the participant of their allocation, and the therapists will be informed about participants allocated to the intervention arm.

All participants will be requested to complete a follow-up questionnaire at 3 months post-randomisation. Participants recruited at least 3 months prior to the end of the recruitment period will also be invited to complete a questionnaire at 6 months post-randomisation. Participants will have the option to receive questionnaires by post or online. Non-responders will be reminded using postal, telephone, online and text prompts.

A clinician may decide that a participant should be withdrawn from the intervention if there is reason to believe they have become unsuitable for the study. Participants can also withdraw from one or more of the following: intervention, data collected from them via questionnaires or data about them from medical records.

### Treatment as usual

All participants will receive treatment as usual which will be the standard care offered to women at this stage of their treatment for cancer. Women will be invited to an in person or remotely delivered end of treatment summary meeting with a breast cancer nurse involving a holistic needs assessment and provision of information relating to local services that can be accessed. Follow-up is patient-initiated, whereby the majority of patients are discharged from hospital follow-up, but are given contact details of a breast cancer nurse that they can speak with if they have any problems or concerns. The breast cancer nurse can make referrals to an oncologist, surgeon or other healthcare professional as needed. The content of treatment as usual received by participants will be collected for all participants in the trial.

### ACTION intervention plus treatment as usual

All participants in this group will receive treatment as usual in addition to the ACTION intervention. The ACTION intervention was co-designed with women affected by breast cancer and healthcare professionals. It is designed to enhance psychological flexibility as a means to improve AET adherence and quality of life, while also being brief, and deliverable without significant changes to NHS services and staffing. The ACTION intervention is comprised of one individual ACT session (1 h) followed by three group sessions (90 min each). Sessions will be delivered by practitioner psychologists remotely using locally approved videoconferencing software and will be digitally recorded, with the agreement of the participant. Within the first session, participants will also be directed to a bespoke ACTION website containing supplementary ACT exercises and additional information regarding HTs and advice on managing treatment side-effects. In addition, a printable participant manual that includes all exercises to be undertaken in the four sessions will be sent to participants.

The ACTION intervention’s format, aims and content are described in Table 2. The initial session is delivered by a practitioner psychologist, and they will be accompanied by a qualified member of staff for the group sessions. Access to home practice exercises will be via the ACTION website and in the participant manual. The manual will be sent to participants after the individual session by the therapists. The group sessions will include between 8 and 10 participants; however they can run with a minimum of three confirmed attendees. A participant will be deemed to have received the intervention if they have attended the individual session and at least one of the three group sessions.

**Table 2:** Format, aims and content of the ACTION intervention

Session	Aim	Indicative content
**Individual session** (60 min)	- Assessment of psychological flexibility - Relationship building - Identify some clinically relevant behaviours - Provide a simple formulation	- Explanation of ACT and the ACTION intervention - Conversations exploring a) ongoing treatment and breast cancer experiences and how challenging experiences are managed b) where participants values might lie - Communication of a psychological flexibility formulation - Highlight the ACTION website including ideas for managing side-effects Home practice - ‘Getting to your core values’ exercise - ‘What do you do with difficult feelings?’ exercise
**Group session 1** (90 min)	- Enhance awareness of thoughts, feelings and actions - Introduce a values-based framework for making decisions - Introduce ways to relate to difficult thoughts and feelings	- Education: normalising emotional responses to challenging events - Discussion of ‘What do you do with difficult feelings?’ exercise - Introducing defusion to support living with challenging thoughts: - Experiential defusion practice, e.g. ‘thoughts as hands’ exercise ‘I am having the thought that … ’ exercise Home practice - Defusion diary: noticing what thoughts tend to draw one in and practicing defusing from them
**Group session 2** (90 min)	- Develop deeper awareness of values - Consider hormone therapy decisions while considering values - Introduce willingness skills	- Reflection on the ‘Getting to your core values’ exercise - Values compass - Personal reflection on: ‘what is the smallest possible step you could do to get in touch with your values’ - Reflection on whether treatment adherence or side-effect management is a values-consistent behaviour - ‘Passengers on the bus’ exercise Home practice Smallest possible step exercise Defusion diary
**Group session 3** (90 min)	- Enhance awareness of getting caught up in unhelpful stories - Introduce ideas for stepping back from stories - Reinforce new useful skills	- ‘Notice who is noticing’ exercise - ‘Letting go of unhelpful labels’ exercise - Reflection on what has and has not been useful over the course of the intervention. Reminder of the website including ideas for managing hormone therapy side effects

The intervention was co-designed prior to the COVID-19 pandemic. We subsequently altered the method for delivering the intervention with support from patient representatives from face-to face delivery in a hospital setting to remote delivery using videoconferencing software. Participants are invited to attend from their own homes, while therapists are based at the hospital site or their home, in line with the standard practice for their clinical service.

Participants allocated to the ACTION intervention will be contacted by the psychology team delivering the treatment sessions. They will also be reminded about the ACTION website and encouraged to look at it prior to the session. The first session of the ACTION intervention will take place within 3 months of randomisation. The first group session will take place within six weeks of the first individual session.

#### Participant website

The provision and content of a website was suggested by attendees of the co-design workshop (39), and designed by the research team with support from patient representatives. It contains sections on side-effect management, patient stories (via HealthTalk.org), ACT resources and exercises, and signposting for further information/support. The CTRU will email participants allocated to the ACTION intervention with log in details to the study specific website prior to the first session.

### Therapist training, competency and fidelity assessment

As a novel intervention, we are particularly interested in designing training to enhance competency, and to assess the extent to which therapists deliver ACT with fidelity and deliver all ACTION study procedures.

#### Therapist training

All therapists will receive training in the ACTION intervention, which was designed and will be delivered by CG and JC. They are both registered clinical psychologists with experience in ACT and supporting women with breast cancer, respectively. Training will be delivered remotely over two full days via video conferencing software. The training will outline the psychological challenges of living with breast cancer and HTs, general ACT principles and the practice of ACTION intervention-specific therapy methods. To ensure consistency in intervention delivery, therapists will be given a comprehensive printable training manual and PowerPoint slides to support the group sessions. To assess whether therapists achieve sufficient knowledge of ACT principles, we will assess knowledge of ACT processes after training [43]. They will be offered fortnightly ACT group supervision (60 min) with CG via video conferencing software, and can also access local clinical supervision (JC) as required.

A pool of therapists will be trained at the central delivery site to accommodate attrition. If a therapist is unavailable, efforts will be made to identify a second therapist from the pool of therapists. If no therapist is available from the pool, an additional therapist will be identified from within the clinical service. Prior to delivering an ACTION session, they will be asked to view a recording of the training sessions, and attend a 1–2 h booster session with CG to ensure competency.

#### Competency

To support the establishment of competency CG will analyse the audio recording of the first individual session of all clinicians delivering the intervention, rate their performance against the ACT Fidelity Measure (ACT-FM) [44] and review the therapist checklist. The therapists will receive prompt feedback, and additional training will be offered where appropriate.

#### ACT fidelity

At the end of study, two independent assessors with expertise in ACT-based psychological interventions will listen to ten randomly selected therapy tapes and rate them for ACT fidelity using the ACT-FM. A minimum of one session will be reviewed from each therapist involved.

#### Procedural fidelity

We will also review therapist self-reports of fidelity to ACTION specific procedures via inspection of the Procedural Fidelity checklist.

### Measures

The measures collected and their timing are illustrated in Tables 3 and 4. Primary and secondary endpoints are described in Table 5.

**Table 3:** Summary of assessments and timing

Assessment	Source	Method of completion	Timeline			
			Screening	Baseline	3 months	6 months (for patients recruited at least 3 months prior to the planned end of recruitment)
**PARTICIPANT**						
Screening	Screening CRF	Research nurse/delegate	X			
Contact details	CRF	Research nurse/delegate		X	CTRU to be notified of address changes	
Demographics including comorbidities (Charlson Comorbidity Index)	CRF	Self-completion/research nurse/delegate		X		
Eligibility (including inclusion and exclusion criteria)	CRF	PI/research nurse/pis delegate		X		
Consent	Consent Form	Self-completion/RN/delegate over the telephone		X		
Randomisation	CRF/CTRU Online system	Research nurse/delegate		X		
ASK-12	Questionnaire	Self-completion		X	X	X
McGill Quality of Life-Revised	Questionnaire	Self-completion		X	X	X
Work and Social Adjustment Scale	Questionnaire	Self-completion		X	X	X
HFRDIS	Questionnaire	Self-completion		X	X	X
MAF	Questionnaire	Self-completion		X	X	X
PROMIS Pain Interference	Questionnaire	Self-completion		X	X	X
DIVA (Part C)	Questionnaire	Self-completion		X	X	X
GAD-7	Questionnaire	Self-completion		X	X	X
PHQ-9	Questionnaire	Self-completion		X	X	X
FACT-ES	Questionnaire	Self-completion		X	X	X
Valuing Questionnaire (VQ)	Questionnaire	Self-completion		X	X	X
UK Cancer Costs	Questionnaire	Self-completion		X	X	X
Acceptability	Questionnaire	Self-completion				X
B & N Questionnaire	Questionnaire	Self-completion				X
Safety Reporting	CRF	Research nurse/PI/delegate/self-completion		X		
Usual Care Data	CRF	Researcher/delegate/self-completion		X	X	X

**Table 4:** Summary of therapist, fidelity and competency assessments and timing

Assessment	Source	Method of completion	TIMELINE								
			Pre-therapist training	Post-therapist training	Baseline	Individual therapy session	1st group session	2nd group session	3rd group session	3 month follow-up	6 months follow-up
**THERAPIST**											
ACTKQ	Questionnaire	Therapist		X							
Demographics	CRF	Therapist			X						
ACT-FM	Questionnaire	Independent Expert Reviewer							X		
Procedural Fidelity Checklist	CRF	Therapist				X	X	X	X		
Clinician competency using ACT-FM*	Questionnaire	ACTION Trainer				X					

^*^Reviewing audiotapes from the first individual session of the nominated lead at each site, the reviewer will complete an ACT-FM form

**Table 5:** Primary and secondary endpoints recorded within the ACTION trial

Primary endpoints	Secondary endpoints
Number of patients screened for eligibility	Intervention adherence for patients in the intervention arm (number of sessions attended, number of participants in group sessions, whether homework was completed, reasons for non-attendance/non-compliance)
Number and proportion of patients eligible out of those screened and reasons for ineligibility	Website use (tracking data to include number of visits, pages visited, materials downloaded, videos watched, clicked links)
Number and proportion of patients consenting to randomisation out of those eligible and reasons for non-randomisation	Number of patients deemed as having completed the intervention
Number of participants randomised per site per month	Treatment as usual content in both arms from participants (number of sessions attended and content of sessions) and sites (initiatives implemented during the trial)
Questionnaire completion rates at each time-point	Safety (deaths, hospitalisations related to the intervention, RUSAEs)
Number of items of missing data per questionnaire at each time-point	Acceptability of the ACTION intervention to participants (acceptability and B&N questionnaires)
Number and proportion of randomised participants lost-to-follow-up	Number, proportion and timing of therapist withdrawals and reasons for withdrawal
Number, proportion, type and timing of participant withdrawals out of those randomised and reasons for withdrawal	Qualitative study
	Competency and fidelity of the delivery of the ACT intervention by NHS therapists (ACTKQ, ACT-FM, Procedural fidelity checklist)
	“Proof-of-principle” exploration in outcome and process variables
	Estimation of the intracluster correlation coefficient

#### Trial data collection

We will collect data related to trial screening, eligibility, consent, contact details and randomisation, questionnaire completion, withdrawal and intervention adherence.

#### Participant measures

##### Participant characteristics

We will collect data on NHS number, date of birth, marital status, employment status, education, menopausal status, number of children, year of diagnosis, stage of cancer at diagnosis, breast cancer type (primary, secondary), breast cancer treatment received, co-morbidities, hormone therapy regimen, supportive therapies used and previous exposure to psychotherapies.

##### Adherence Starts with Knowledge (ASK)-12 [45]

A 12-item, patient-report measure of barriers to medication adherence and adherence-related behavior. This is the planned future primary outcome in any future phase III trial.

##### McGill Quality of Life-Revised [46]

A 14-item, patient-report tool designed to measure physical well-being, physical symptoms, psychological symptoms, existential well-being and support, as well as overall quality of life of people with life-threatening illness.

##### Work and Social Adjustment Scale [47]

A 5-item self-report measure looking at how a specific illness/disorder impacts patient ability to function day to day with depression and/or anxiety as well as phobic disorders.

##### Hot Flash Related Daily Interference Scale (HFRDIS) [48]

A 10-item self-report questionnaire used to assess the impact of hot flashes on daily life and overall quality of life following breast cancer.

##### Multidimensional Assessment of Fatigue (MAF) [49]

A 16-item self-report scale that measures fatigue according to 4 dimensions; degree and severity, distress that it causes, timing of fatigue, and its impact on various activities of daily life.

##### PROMIS Pain Interference [50]

A 6-item self-report questionnaire measuring the consequences of pain over the last 7 days on relevant aspects of a person’s life, including engagement with social, cognitive, emotional, physical and recreational activities.

##### Day-to-Day Impact of Vaginal Aging Questionnaire: DIVA (part C) [51]

Part C (scale focusing on sexual functioning) of the DIVA questionnaire contains 8 self-report items relating to the impact of vaginal symptoms on functioning and well-being.

##### Generalised Anxiety Disorder questionnaire (GAD-7) [52]

The GAD-7 is a 7-item self-report anxiety questionnaire designed to assess the patients’ health status during the previous 2 weeks.

##### Patient Health Questionnaire-9 (PHQ-9) [53]

The PHQ-9 is a 9-item self-reported scale providing a valid and reliable measure of depression severity.

##### Functional Assessment of Cancer Therapy–Endocrine Symptoms [54]

The FACT-ES is a self-report quality of life questionnaire for people with cancer, consisting of 4 subscales which examine physical well-being with 7 items, social/family wellbeing with 7 items, emotional wellbeing with 6 items and functional wellbeing with 7 items as well as a series of items relating to additional concerns.

##### Valuing Questionnaire (VQ) [55]

A 10-item self-report questionnaire to measure aspects of psychological flexibility—mainly how consistently an individual has been living commensurate with their self-determined values.

##### Acceptability

Those participants recruited 3 months prior to the end of the recruitment phase and randomised to the ACT–based intervention will also complete items related to the acceptability of the intervention at 6 months post-randomisation only. Items ask about the acceptability of the different components of ACTION and whether participants found it useful.

##### Borkovec and Nau Acceptability Questionnaire (B&N) [56]

Three items adapted from the B&N will be collected, based on the wording used in the OBI study [57]. This is an additional measure of acceptability and will be used within the progression criteria.

##### UK Cancer Costs Questionnaire [58]

We adapted the UK Cancer Costs Questionnaire to assess services accessed by the participants at baseline and follow-up time points.

#### Therapist measures

##### ACT Knowledge Questionnaire (ACTKQ) [43]

The ACTKQ is an assessment of therapist knowledge of the principles of ACT. It has 16 items.

##### Procedural fidelity checklist

The procedural fidelity checklist was designed for the ACTION trial for therapists to self-rate whether or not they undertook crucial intervention procedures that were previously outlined in training and in the manual. It has 26 items, and is rated at the end of each of the four sessions.

##### ACT Fidelity Measure (ACT-FM) [44]

The ACT-FM is a measure of therapist fidelity to ACT principles when delivering treatment. There are 25 items capturing pre- and pro-scribed therapist behaviours when delivering ACT. The ACT-FM is scored when reviewing the therapy tapes of a clinician delivering ACT.

#### Site measures

##### Intervention adherence

Intervention adherence data will be collected from the delivering site and will include the number of sessions attended and the number of participants in the group sessions.

##### Treatment as usual monitoring

Treatment as usual will be monitored at all recruitment sites. It will include data at the service level, including services offered, to allow for an assessment of treatment as usual. This data will be collected at the end of recruitment with sites asked to report treatment as usual throughout the recruitment phase.

### Sample size

As the study is randomised and we wish to obtain a preliminary estimate of effectiveness in relation to demonstrating how the intervention affects medication adherence, we have used methods developed for phase II screening trials in oncology [59] in which preliminary and non-definitive randomised treatment comparisons are made, carefully adjusting the false-positive (a) and false-negative (ß) error rates so the target treatment effect is appropriate while the sample size remains restricted. Allowing for 25% loss to follow-up and using a 1-sided *t* test with a significance level of 20% will allow us to detect an effect size of 0.432 with 65% power using a sample of 80 patients. The sample size must be sufficient to establish consent and dropout rates (Objective i) and test trial protocols and acceptability (Objectives ii–iii). A sample size of *N* = 40 per arm will be sufficient to meet these aims [60–62].

### Nested qualitative study

Following completion of the study, all participants assigned to the intervention group will be invited to participate in telephone interviews with a researcher. Interviews will focus on the acceptability of the intervention, trial procedures and outcome measures. We will seek to identify components of the intervention that were perceived as particularly helpful or unhelpful to participants. If aspects of the trial are considered unacceptable or unhelpful, questions will be asked to resolve some of these issues in preparation for a phase III RCT.

The therapists delivering the intervention will also be invited to participate in semi-structured telephone interviews. These will inquire about therapist’s experiences of delivering the intervention, the practicability of the intervention (within NHS contexts), training and ways to improve the programme.

### Analysis

A detailed statistical analysis plan will be written before any analysis is undertaken. The analysis will focus on descriptive statistics and confidence interval estimation rather than formal hypothesis testing and no formal evaluation of safety or efficacy of the study interventions will be conducted. All analyses will be undertaken on the intention-to-treat population, with all participants included in the analysis according to their randomised allocation, and regardless of non-adherence to the intervention or withdrawal from the study. Final analysis will be conducted once all available outcome data is received.

Descriptive summaries will be presented overall, by arm and by site (where relevant) using appropriate frequencies and summary statistics. Outcome measures will be scored according to relevant scoring manuals. To generate evidence of proof of principle, the mean change from baseline in the three and 6-month ASK-12 scores in both arms will be reported, together with a range of confidence intervals around the main estimate to inform us as to the likelihood of where the ‘true’ estimate may lie. Analysis will adjust for the minimisation factors.

The trial is not powered to provide a precise estimate of the level of clustering relating to therapist effects, but we may be able to investigate this effect. If a sufficient number of therapists are involved in delivering the intervention, we will estimate the intraclass correlation coefficient and produce a range of confidence intervals (e.g. 95%, 67% and 51%) around this to inform the sample size of the definitive trial.

The semi-structured interviews will be analysed using thematic analysis [63].

### Progression criteria

Progression criteria will be used to judge whether it is feasible to progress to a larger study (Table 6). Analysis of progression criteria will take place during final analysis of the trial.

**Table 6:**
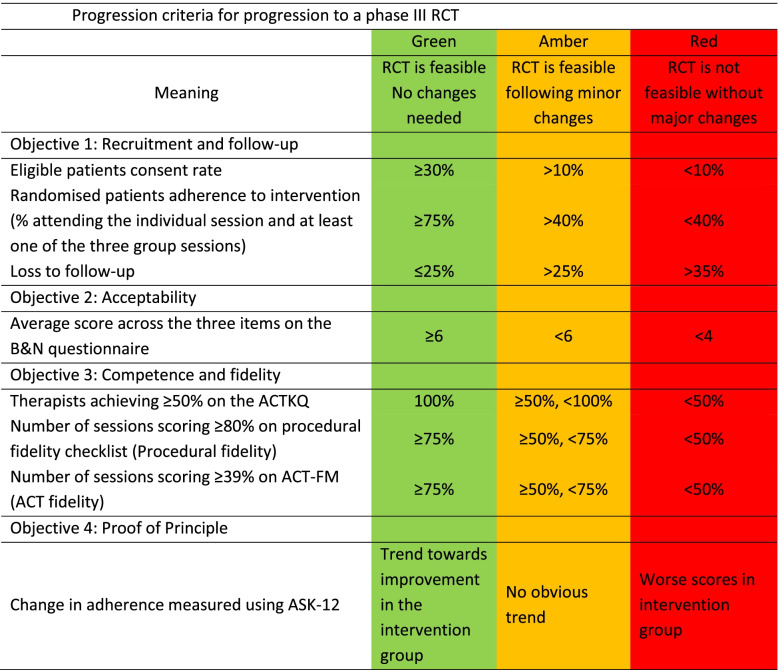
Progression criteria for progression to a phase III RCT

### Data management

Data collection forms transferred to or from the CTRU will be coded with a study number (made up of the recruitment site code and the participant’s unique sequential trial number), the participant’s initials, and date of birth. Study data will be held securely on paper and electronically at the University of Leeds’ CTRU, and appropriate processes put in place for the transfer, storage, restricted access, and disposal of personal information. Relevant Standard Operating Procedures, Guidelines and Work Instructions in relation to data management, processing, and analysis of data will be followed.

### Access to data

Data will only be shared for participants who have given consent to use of their data for secondary research, and will only be made available in such a way that recipients cannot identify individuals by any reasonable likely means, and we will only share data for projects that are clearly in the public interest and compatible with the original purpose of the data processing. Requests to access trial data should be made to CTRU-DataAccess@leeds.ac.uk in the first instance.

### Independent monitoring

The Trial Steering Committee has responsibility for the oversight of the trial, including trial progress, adherence to protocol, participant safety and consideration of new information. The trial steering committee will be provided with reports prepared by the CTRU. It has been formed and consists of an independent chair (statistician), a clinical psychologist, a behavioural scientist and two patient representatives. The Committee will meet annually as a minimum. As this is a pilot study and the level of risk involved is low with regard to patient safety there will be no data monitoring committee convened. The Trial Steering Group will adopt a safety function where it becomes necessary.

### Safety

In this population, we expect episodes of acute illness, infection, new medical problems and deterioration of existing medical problems to occur. These could result in prolonged hospitalisation, hospital re-admission, significant or permanent disability or incapacity, or death. In recognition of this, events fulfilling the definition of a serious adverse event will not be reported in this study unless the event resulted from administration of any research procedure and fulfils definition of a related and unexpected serious adverse event (RUSAE). Deaths will be recorded, but not subjected to expedited reporting. Hospitalisations will be recorded by contacting sites at the end of the study. Pregnancies will also be reported to the CTRU.

All RUSAEs occurring from the date of consent until the end of researcher contact will be reviewed by the Chief Investigator and be subject to expedited reporting to the Sponsor and the main research ethics committee by the CTRU on behalf of the CI within 15 days.

Concerns over patient safety will be monitored by reviewing responses to pre agreed ‘red flags’ assigned to the PHQ-9 questionnaire (if question 9 is answered 1–3 at any time point). If this occurs in the baseline questionnaire, the Chief Investigator (or delegate) will be notified within 2 working days of receipt at the CTRU and will contact the patient to follow up and repeat the screening questionnaire with them to make sure they are (a) not a danger to themselves or others, and (b) still eligible. If this response is given during the follow-up questionnaires the Chief Investigator (or delegate) will be notified within 2 working days of receipt at the CTRU and will report this to the PI at the recruiting site. If the patient is still receiving trial treatment, the psychology team at the central delivery site will be informed and will contact the patient and repeat the screening questionnaire as above.

## Discussion

Non-adherence to AET is a major problem among women with breast cancer [4] and is linked with an increased risk of cancer-specific and all-cause mortality [5–12]. Women who are unable to adhere to their adjuvant treatment are likely to need additional support to address the challenges they experience with this behaviour, and with their broader quality of life. A number of barriers to medication use have been reported in this setting, including medication side-effects, psychological distress and a lack of social support [4, 15]. The ACTION intervention aims to address these barriers through a co-developed remotely delivered group-based psychotherapy programme, informed by ACT.

Prior to testing the ACTION intervention in a phase III RCT, additional information is needed across a range of areas. This includes exploring the acceptability of the intervention to participants and NHS staff, examining the extent to which NHS therapists can deliver the intervention with fidelity, and demonstrating the feasibility of recruiting and retaining patients in this setting. This exploratory multi-centre pilot trial with a nested qualitative study aims to provide information to inform our future decision as to the feasibility of a definitive trial. While we are not attempting to evaluate the efficacy of the intervention, it will be our first opportunity to examine signals of efficacy of the intervention on our planned outcome measures.

The ACTION intervention advances our knowledge of ACT as it is among the first programmes to be tested within an RCT to focus on medication decision-making and quality of life. Although ACT has been proposed as a means to enhance adherence to treatments, and case series have emerged in recent years, there have been few comprehensive assessments of the approach [29, 37, 38]. Also novel within the methodology of ACTION is the comprehensive focus on therapist competency and fidelity to ACT principles and ACTION procedures. For psychological interventions that require skill in implementation, this pilot offers an important opportunity to understand the competency and fidelity of delivering the intervention. This information will be used to improve training, supervision and the suitability of the intervention for clinical practice.

In response to the COVID-19 pandemic, our intervention will involve remotely delivered therapy, using video conferencing software. This alteration to original plans brings new opportunities with regard to widespread implementation and uptake. Participants do not need to travel to attend and capacity is not limited by the availability of rooms within hospitals. We anticipate that it may also increase access to specific groups who may be unable to access in-person support as readily, including rural populations, those lacking transport links, and younger populations who have returned to work following treatment. A remotely delivered approach may also have limitations, some of which are beginning to emerge following evaluations of clinical care during the COVID-19 pandemic [64]. Potential problems include difficulties building therapeutic relationships as easily, technical challenges in using unfamiliar software and hardware, and the possibility of disenfranchising women who cannot access the internet or a private space to participate fully within groups. These issues will be carefully considered using the data provided within the pilot trial, and may be most apparent within the nested qualitative study.

In conclusion, this multi-centre randomised controlled external pilot trial will examine the feasibility of delivering a co-developed psychotherapy intervention to support medication adherence and quality of life in women with breast cancer. If the pilot data indicate it is feasible, ACTION can be evaluated in a larger phase III RCT.

### Study status

#### Enrolling

Recruitment began 04/2021. Approximate date recruitment will be completed: 09/2022. Latest protocol version approved 5.0_030221 on 05/02/2021 by Yorkshire & The Humber–South Yorkshire Research Ethics Committee (20/YH/0104).

### Study amendments

Not applicable.

## Data Availability

Not applicable.

## Appendix

Appendix 1: The TIDieR (Template for Intervention Description and Replication) Checklist: Information to include when describing an intervention and the location of the information

**Table:**

N^**o**^	What	Details
**1**	**Name**	The Yorkshire Cancer Research ACTION Pilot Trial: An ACT-based intervention for women with breast cancer to support wellbeing and hormone therapy medication decisions
**2**	**Why: Rationale, theory, goal**	Adjuvant hormone therapies are prescribed at the end of hospital-based breast cancer treatment in order to prevent recurrences and all-cause mortality. However, adherence to these medications is often poor, due in part to intolerable side-effects. This time during the cancer journey is also particularly challenging psychologically, as women are transitioning from ‘patient’ to ‘survivor’. They also report a lack of support during this time, post hospital discharge. Previous adherence interventions have focussed on information giving and/or problem-solving difficulties with medication-taking (e.g. disorganised regimens), and have had little effect. Given the wide range of additional emotional (e.g. mood, motivation) and somatic (e.g. side effects) factors that contribute to low adherence, it is perhaps unsurprising that these previous interventions have been unsuccessful. An alternative, and potentially more effective strategy, is to design an intervention to target a range of emotional and physical factors that may affect adherence. Furthermore, using co-design methods may be particularly beneficial, to ensure that any proposed intervention is acceptable to patients, as well as feasible to implement within routine NHS care. Acceptance and Commitment Therapy (ACT), is potentially well suited to tackle this problem, and has been show to improve outcomes in those living with chronic illness, chronic pain, and cancer. ACT is particularly suited to helping people function effectively in objectively difficult situations (e.g. living with illness or immutable pain). ACT aims to increase a participant’s awareness of their personal values, and to undertake more of the behaviours that support these values – a process that often involves developing a willingness to have painful thoughts and feelings (such as medication side-effects). Given the above, we have co-designed and conducted a small run-through of an ACT intervention for women with breast cancer who have been prescribed adjuvant hormone therapies. The aim of the intervention is to support wellbeing, and hormone therapy medication decisions and adherence. This pilot RCT aims to feasibility test this intervention.
**3**	**What Materials**	Practitioner psychologists delivering the intervention received two days of bespoke training delivered by clinical psychologists with ACT and cancer expertise. Alongside this, they received a training manual, with information about ACT generally, and specific session plans for the intervention sessions. Participants received an intervention manual, containing the session plans, tasks, and homework tasks for in-between sessions.Participants also had access to a bespoke website, containing additional ACT exercises and information, strategies for self-management of side-effects, and signposting to further sources of support.
**4**	**What Procedures**	*Clinician Training* (See section 5) *Intervention delivery* 1x Individual session with a practitioner psychologist delivered remotely via a video platform 3x Group sessions with a practitioner psychologist delivered remotely via a video platform ACT tasks to practice at home between sessions Website to access additional resources *Evaluation of the Clinician Training* ACT Knowledge questionnaire administered post-training Clinician fidelity evaluated using the ACT-FM completed by an external rater Clinician fidelity to intervention procedures will involve clinician self-rating using a procedural fidelity checklist. Clinician competency will be assessed by Dr Graham, using the ACT-FM, through reviewing audiotapes from the first individual session of all therapists at the treatment delivery site. *Evaluation of the Intervention* Adherence, Quality of Life / Symptom Burden, Anxiety & Depression measured pre- and post- intervention. Acceptability of the intervention. Website use tracked. *Support activities* Recruitment and consent of participants
**5**	**Who provided**	The practitioner psychologists delivering the intervention underwent training in delivering Acceptance and Commitment Therapy. The training was delivered by Dr Chris Graham (CG), who has expertise in ACT applied to chronic disease, and Dr Jane Clark (JC), a consultant clinical psychologist working in oncology at St. James University Hospital. Training included teaching about ACT and practice of intervention-specific therapy methods. This course consisted of two full days of training along with set tasks to complete at home, equating to approximately 5 more hours of home practice. Each site’s psychologists had a varied background that may or may not have included previous ACT training prior to our delivered training programme. However, all session leads were Health and Care Professional Council (HCPC) registered practitioner psychologists (Clinical, Health or Counselling Psychologist) who worked with breast cancer patients in a hospital setting.
**6**	**How: mechanisms of delivery**	The individual session will be delivered remotely via video platform by the psychologist. The group sessions (3 in total) will be delivered in a group of 8-10 participants, remotely via video platform, also by a psychologist (and a supporting member of staff). Home practice tasks will be delivered via the paper participant manual, but they could also be accessed via the bespoke website. Participants will be directed to check out the additional information (on side-effect management, and extra ACT resources) on the website, which contains written information, audio clips, and video clips.
**7**	**Where: location of delivery**	All sessions will be delivered remotely via an on line video platform by a central delivery team at Leeds Hospitals NHS Trust.
**8**	**When and how much**	The individual session will last for 60 minutes. Within six weeks of this session, the first group session will take place. All 3 group sessions will last 90 minutes and will be held every two weeks.
**9**	**Tailoring**	Although there is a set session plan to follow, detailing specific exercises and tasks for each session, the therapy itself is quite flexible. As such, the deliverer may adapt the content to ensure it’s relevant to each participant (e.g. through discussing specific individuals’ values, goals, and behaviours).
**10**	**Modifications**	*<To be completed post study completion>*
**11**	**How well (planned)**	Clinician fidelity to competently deliver the intervention in line with ACT will be assessed by an external rater with a background in ACT. They will complete the ACT-FM checklist while listening to the audio recording of ten sessions. A score of above 28 (out of 72) is considered competent. Additionally, an intervention specific metric of “Procedural Fidelity” is included, which measures other aspects of the intervention that are important for treatment fidelity but are not ACT-specific (e.g. giving worksheets, presenting information etc.). It includes 7 items for session 1 (individual session), 10 items for the first group session, 9 items for the second group session, and 8 items for the third group session. A percentage score is created for each session by dividing the score achieved by the maximum possible score achievable within that session, and multiplying by 100.
**12**	**How well (actual)**	*<To be completed post study>*

## Abbreviations

ACT: Acceptance and Commitment Therapy
AET: Adjuvant endocrine therapy
ACT-FM: ACT Fidelity Measure
ACTKQ: ACT Knowledge Questionnaire
ACTION: Acceptance and Commitment Therapy Intervention
ASK: Adherence Starts with Knowledge
AIs: Aromatase inhibitors
B&N: Borkovec and Nau
CTRU: Clinical Trials Research Unit
COVID-19: Coronavirus disease-19
DIVA: Day-to-Day Impact of Vaginal Aging Questionnaire
FACT-ES: Functional Assessment of Cancer Therapy – Endocrine Symptoms
GAD-7: Generalised Anxiety Disorder questionnaire
HFRDIS: Hot Flash Related Daily Interference Scale
HCPC: Health and Care Professional Council
HIV: Human immunodeficiency virus
MAF: Multidimensional Assessment of Fatigue
NHS: National Health Service
NRES: National Research Ethics Service
ER+: Oestrogen receptor positive
PHQ-9: Patient Health Questionnaire- 9
RCT: Randomised controlled trial
RUSAE: Related and unexpected serious adverse event
SERMs: Selective Oestrogen Receptor Modulators
VQ: Valuing questionnaire
WSAS: Work and Social Adjustment Scale
